# Equine coronavirus infection and replication in equine intestinal enteroids

**DOI:** 10.1186/s13567-024-01381-z

**Published:** 2024-10-10

**Authors:** Yoshinori Kambayashi, Manabu Nemoto, Akihiro Ochi, Daiki Kishi, Takanori Ueno, Koji Tsujimura, Hiroshi Bannai, Nanako Kawanishi, Minoru Ohta, Tohru Suzuki

**Affiliations:** 1 Equine Research Institute, Japan Racing Association, 1400-4 Shiba, Shimotsuke, Tochigi 329-0412 Japan; 2 Division of Zoonosis Research, Sapporo Research Station, National Institute of Animal Health, 4 Hitsujigaoka, Toyohira, Sapporo, Hokkaido 062-0045 Japan

**Keywords:** Equine coronavirus, equine intestinal enteroid, horse, viral infection

## Abstract

In this study, equine intestinal enteroids (EIEs) were generated from the duodenum, jejunum, and ileum and inoculated with equine coronavirus (ECoV) to investigate their suitability as in vitro models with which to study ECoV infection. Immunohistochemistry revealed that the EIEs were composed of various cell types expressed in vivo in the intestinal epithelium*.* Quantitative reverse-transcription PCR (qRT-PCR) and virus titration showed that ECoV had infected and replicated in the EIEs. These results were corroborated by electron microscopy. This study suggests that EIEs can be novel in vitro tools for studying the interaction between equine intestinal epithelium and ECoV.

## Introduction, methods and results

Equine coronavirus (ECoV) primarily infects the small and large intestines of horses, resulting in fever, anorexia, lethargy, and diarrhea [1, 2]. Most affected horses exhibit only mild clinical signs, but in rare instances, ECoV causes fatal enteritis with severe watery diarrhea or neurological disorders due to hyperammonemia [3, 4]. In most in vitro studies of ECoV infection, HRT-18G cells derived from human rectal adenocarcinoma have been used, because there is no cell line derived from equine intestinal epithelium [5–7]. Although HRT-18G cells support ECoV replication, they should not fully mimic the physiological properties of horses in vivo because this cell line is derived from human cells. Therefore, it is desirable to develop an in vitro system that can closely recapitulate the equine intestinal epithelium for further research on ECoV pathogenesis, including the elucidation of the primary target cells and the immune response to viral infection. Intestinal enteroids have recently been developed as novel in vitro models with which to investigate host–virus interactions in humans and animals [8–11]. They are derived from undifferentiated stem cells located in the crypt bases of the intestines with the capacity for self-renewal and multipotency [12]. They construct three-dimensional (3D) structures composed of various cell types expressed in vivo in the intestinal epithelium and recapitulate physiological activity of the intestinal epithelium in vivo [13]. Recently, equine intestinal enteroids (EIEs) have been established from equine jejunal tissues and used to evaluate immune responses [14–16]. However, to our knowledge, there has been no report describing the infectivity of enteric viruses in EIEs. Elucidating the infectivity and proliferative capacity of ECoV in EIEs indicate that they can be useful in vitro models for studying ECoV infection, which will advance our understanding of the pathogenesis of ECoV infection. In this study, we generated EIEs from the small intestinal tissues of the duodenum, jejunum, and ileum, and investigated the potential for ECoV infection and replication in these EIEs.

Intestinal crypts were harvested from a 3-year-old Thoroughbred horse without gastrointestinal diseases that had been euthanized for reasons unrelated to this study. Crypts were isolated according to a protocol for mouse and human tissues, with some modifications [13]. In brief, approximately 3-cm each of the duodenum, jejunum, and ileum tissue were resected immediately after euthanasia. They were chopped into 5-mm pieces and washed vigorously in ice-cold Dulbecco’s phosphate-buffered saline (D-PBS) ( −) (FUJIFILM Wako Pure Chemical Corporation, Osaka, Japan) until the supernatant became clear. The fragments were incubated in ice-cold D-PBS ( −) containing 5 mM EDTA on ice for 30 min and vigorously pipetted to detach the crypts from the epithelium. The supernatant was filtrated through a 100-μm cell strainer to remove villi. The filtrate was then centrifuged for 5 min at 400 × *g* at 4 °C and the pelleted crypts were resuspended in 25 μL of ice-cold Matrigel (Corning, NY, USA). Matrigel containing the crypts was dropped onto a pre-warmed 48-well plate (Thermo Fisher Scientific, MA, USA). For tissue culture, Advanced DMEM/F-12 medium containing 10 mM HEPES, 1 × GlutaMAX (all from Thermo Fisher Scientific), and a mixed solution of 100 units/mL of penicillin and 100 μg/mL of streptomycin (Nacalai Tesque, Inc., Kyoto, Japan) was prepared as a basal medium. The following growth factors were added to the basal medium to create expansion medium for the maintenance of the stem cell niches and for cell proliferation: 1/10 Afamin/Wnt3a CM (MBL, Tokyo, Japan); 100 ng/mL Noggin (Peprotech, NJ, USA); 500 ng/mL R-spondin, 500 nM A83-01 (both from R&D Systems, MN, USA); 1 × B27, 1 × N2 supplement, 10 nM Gastrin I, 50 ng/mL mouse recombinant EGF protein, 10 μM Y-27632,10 μM SB202190 (all from Thermo Fisher Scientific); 1 mM N-acetylcysteine (FUJIFILM Wako); and 2.5 μM CHIR (Cayman Chemical, MI, USA). Following the polymerization of the Matrigel for 15 min at 37 °C, 250 μL of expansion medium was added and the plates were incubated at 37 °C in 5% CO_2_. Expansion medium was refreshed every 3–4 days and EIEs were passaged at intervals of approximately 5–8 days. After the overlaid medium was removed, the Matrigel domes were mechanically disrupted with ice-cold basal medium. The resuspended EIEs were further dissociated and centrifuged for 5 min at 400 × *g* at 4 °C. The pelleted EIEs were resuspended in Matrigel and cultured.

To confirm that the EIEs contained the various cell types expressed in vivo in equine intestinal epithelium, we evaluated them by immunohistochemistry (IHC) and Alcian Blue Periodic Acid Schiff (AB-PAS) staining. EIEs were recovered from the Matrigel and solidified with iPgell (GenoStaff, Tokyo, Japan) according to the manufacturer’s instructions, fixed with formalin. After embedding in paraffin, 3-μm sections of the EIEs were stained with Histofine Simple Stain MAX PO Multi (Nichirei Biosciences, Tokyo, Japan). Primary antibodies were raised against villin (1:2, IR076, Agilent Technologies) for enterocytes, chromogranin A (CgA) (1:2000, 1-28, Yanaihara, Shizuoka, Japan) for enteroendocrine cells, Lysozyme (LYZ) (1:10, Nichirei Biosciences) for Paneth cells, Sox9 (1:4000, AB5535, Millipore, MA, USA) for stem cells, and Ki67 (1:500, ab15580, Abcam, Cambridge, UK) for proliferative cells. Following automated deparaffinization, heat-antigen retrieval was performed. The sections were incubated with the primary antibodies diluted with Antibody Diluent (Abcam) for 1 h at room temperature. Antibody binding was visualized by the addition of 3,3'-diaminobenzidine (Abcam) according to the manufacturer’s protocol. Mucus secreted by goblet cells was stained with AB-PAS. Jejunal tissues of a healthy horse were also stained as positive controls, and tissues incubated without the primary antibody were used as negative controls.

For virus inoculation to evaluate the susceptibility of EIEs to ECoV, the proliferated 3D EIEs were transferred to 2D monolayers on a 48-well flat-bottomed plate. The bottom of the plates had been pre-coated with 200 μL of 2.5% Matrigel diluted with D-PBS ( −) at 37 °C for 90 min. The EIEs recovered from the Matrigel were centrifuged and resuspended in 1 mL of TrypLE Express (Thermo Fisher Scientific). Following incubation at 37 °C for 10 min, the cells were further pipetted for complete dissociation into single cells, and basal medium was added to halt the enzyme reaction. The suspension was centrifuged, and the cells were resuspended in expansion medium. After removal of the Matrigel solution, the dissociated cells were seeded at 1 × 10^4^ cells/cm^2^ and incubated at 37 °C in 5% CO_2_. The expansion medium was refreshed the following day, and the monolayer culture was maintained for an additional 2–3 days until use.

Monolayer EIEs that reached > 80% confluency were used in the virus inoculation test. ECoV strain NC99, isolated from a diarrheic Arabian foal [5], was used. The monolayer EIEs were washed once with 100 μL of basal medium and inoculated with ECoV at 1 × 10^4^ median culture tissue infectious dose (TCID_50_)/well. After incubation for 60 min at 37 °C in 5% CO_2_, the cells were washed twice with 100 μL of basal medium, and 250 μL of expansion medium was added. The overlaid medium samples were collected from three wells at 1, 6, 12, 24, 48, and 72 h post-inoculation (hpi) and subjected to quantitative reverse-transcription PCR (qRT-PCR) to assess viral replication. For evaluation by electron microscopy, dissociated jejunal EIEs seeded at 1 × 10^4^ cells/cm^2^ in a Matrigel-precoated 24-well plate were inoculated with NC99 at 2 × 10^4^ TCID_50_/well. To compare the viral replication rate between EIEs and HRT-18G cells, HRT-18G cells were inoculated with ECoV. The cells were seeded the day prior to virus inoculation on a 48-well flat-bottomed plate at 1.1 × 10^5^ cells/cm^2^ and inoculated with ECoV at 1 × 10^4^ TCID_50_/well. The inoculated HRT-18G cells were maintained in Minimum Essential Medium (MEM; MP Biomedicals, CA, USA) supplemented with 2% fetal bovine serum (FBS; Bovogen Biologicals, Australia), a mixed solution of 100 units/mL of penicillin and 100 μg/mL of streptomycin, and 1 mM sodium pyruvate (Sigma-Aldrich Co., MO, USA). The overlaid medium was collected at 1, 24, 48, and 72 hpi, and the viral copy number was quantified by qRT-PCR.

ECoV RNA in the collected culture medium was quantified by qRT-PCR. Total RNA was extracted in magLEAD 12gc automated nucleic acid extraction system (Precision System Science, Japan). qRT-PCR was performed on a StepOnePlus qPCR system (Applied Biosystems, CA, USA), using TaqPath 1-Step RT-qPCR Master Mix (Thermo Fisher Scientific). We used specific primer sets (ECoV-380f 5′-TGG GAA CAG GCC CGC-3′ and ECoV-522r 5′-CCT AGT CGG AAT AGC CTC ATCAC-3′) and TaqMan MGB probe (ECoV-436p 5′-6-FAM-TGG GTC GCT AAC AAG-MGB-3′) (Thermo Fisher Scientific) to detect the nucleocapsid (*N*) gene of ECoV [17]. The thermal cycling conditions were an initial hold at 25 °C for 2 min, 50 °C for 15 min and 95 °C for 2 min, and then 40 cycles of 95 °C for 3 s and 60 °C for 30 s. All tests were performed in triplicate for each sample, and the average copy numbers were determined.

To determine the viral infectivity of the supernatant samples collected from virus-inoculated EIEs and HRT-18G cells, the viral titers were determined using HRT-18G cells in TCID_50_ method. HRT-18G cells were seeded at 5.5 × 10^4^ cells/well on a flat-bottomed 96-well plate (AGC Techno Glass Co., Japan) the day prior to the test. The cells were inoculated with 100 µL/well of supernatant samples tenfold serially diluted with MEM containing 2% FBS from 10 to 10^6^-fold. The culture was maintained for 5 days at 37 °C in 5% CO_2_. Based on the emergence of cytopathic effects (CPE), the viral titers (TCID_50_/mL) were determined by the Reed and Muench method [18].

For electron microscopy, the jejunal EIEs inoculated with ECoV were fixed with 2% paraformaldehyde and 2% glutaraldehyde in 0.1 M phosphate buffer (PB) (pH 7.4) at 72 hpi. They were then postfixed with 2% osmium tetroxide in 0.1 M PB at 4 °C for 1 h, and embedded in Quetol-812 (Nisshin EM Co, Japan). Ultrathin sections were stained with 2% uranyl acetate and Lead stain solution and examined with a transmission electron microscope (JEM-1400Plus; JEOL Ltd., Japan). The post fixation and subsequent processes were performed by Tokai Electron Microscopy, Inc. (Aichi, Japan).

**Figure 1:**
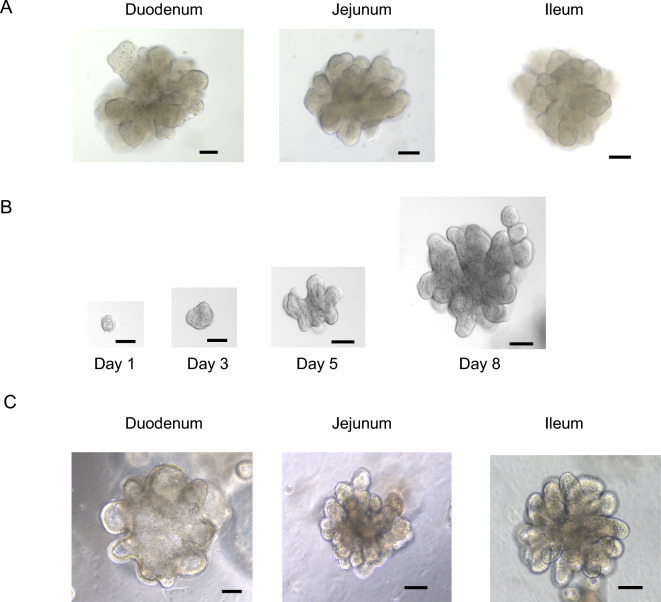
**Microscopy images of EIEs**. **A** Enteroids at 10 days’ culture after isolation of crypts. **B** Representative images of the time course of ileal enteroids growth. **C** Duodenal and jejunal enteroids that were passaged 15 times and ileal enteroids that were passaged 17 times. Scale bar = 100 μm.

**Figure 2:**
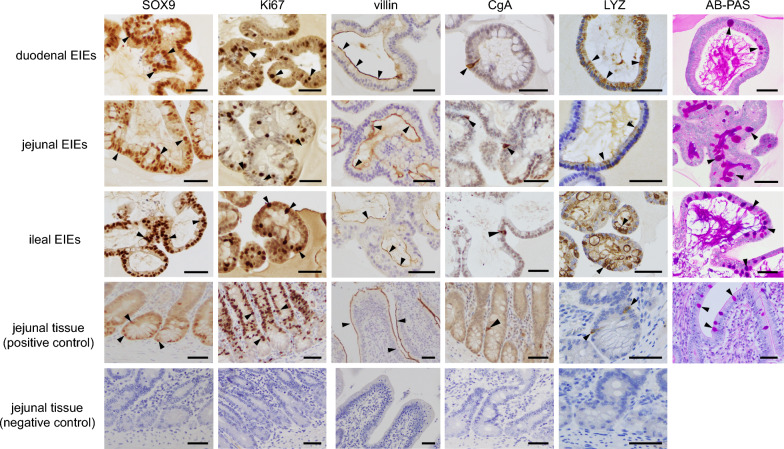
**Identification of the cell types in EIEs by immunohistochemistry and AB-PAS staining**. EIEs derived from duodenal, jejunal, and ileal tissues, as well as jejunum from a healthy horse for positive control, were immunostained (shown as brown) with Sox9 as a cell marker of intestinal stem cells, Ki-67 for proliferative cells, villin for enterocytes, chromogranin A (CgA) for enteroendocrine cells, and lysozyme (LYZ) for Paneth cells. Mucin was stained by AB-PAS as a marker of goblet cells (magenta). Black arrow heads indicate positive stained cells. Sox9-positive cells were identified throughout the EIEs. The negative control samples were not applied with primary antibodies. Scale bar = 50 μm.

Crypts isolated from the duodenum, jejunum, and ileum started to form bud structures after 6–7 days of the culture (Figure 1A). After further development over another 2–3 days, they formed more complex and multilobular structures. The passaged EIEs formed multilobular structures and reached a significant size by days 5–8 (Figure 1B). The optimal split ratio was between 1:6 and 1:10, varying with the number and size of the EIEs. The EIEs could be passaged at least 15 times without visible morphological changes or a decline in proliferation (Figure 1C). IHC showed that a substantial number of Sox9-positive cells were observed throughout the EIEs, as well as Ki-67 positive proliferative cells (Figure 2). Villin-positive enterocytes were observed throughout the luminal surface of the EIEs. In addition, CgA-positive enteroendocrine cells, LYZ-positive Paneth cells, and AB-PAS-positive goblet cells were identified. These cell types were also identified in the tissue sections of healthy jejunum, but no positive cells were observed in negative control samples (Figure 2).

**Figure 3:**
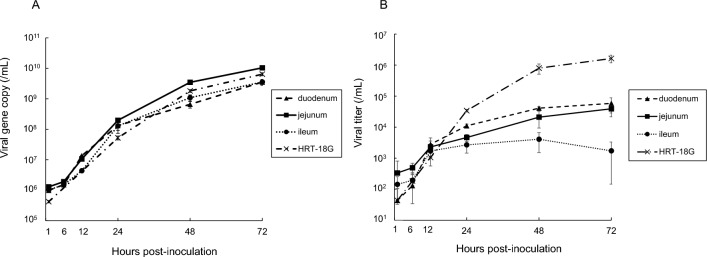
**Active ECoV replication in EIEs and HRT-18G cells**. **A** The ECoV RNA copy number in the culture medium of virus-inoculated EIEs and HRT-18G cells was determined by qRT-PCR. **B** The viral titers in the same samples were determined using HRT-18G cells by the TCID_50_ method. The number of viral genes and the viral titers in 1 mL of the original sample were calculated. Data are presented as the means ± SD.

**Figure 4:**
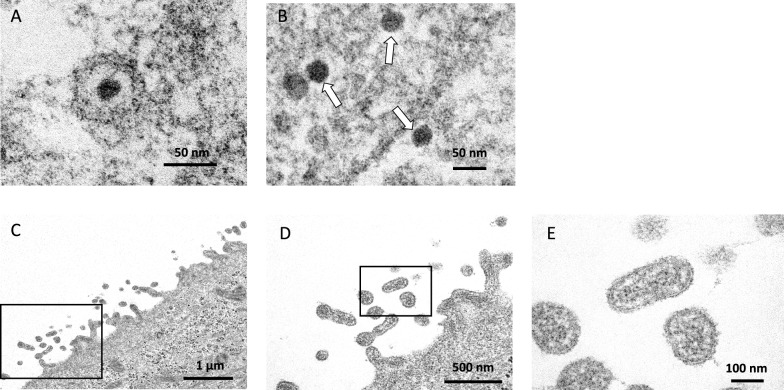
Electron microscopy images of the jejunal EIEs inoculated with ECoV. Jejunal EIEs inoculated with ECoV were fixed and evaluated by electron microscopy. Virus particles were observed in the **A** cytoplasmic vesicles and **B** cytoplasm, and **C**–**E** to be budding from the surface of the cell membrane and releasing into the extracellular space. **D** is a magnified image of (**C**). **E** is a magnified image of (**D**). Scale is shown in each image.

The copy number of ECoV RNA had started to increase at 6 hpi and continued to increase up to 72 hpi in monolayer EIEs (Figure 3A). At 72 hpi, the copy numbers had increased relative to that of 1 hpi by 3563 × in the duodenal EIEs, by 8043 × in the jejunal EIEs, and by 3520 × in the ileal EIEs. No CPE was observed in any of the EIEs. The viral copy number of samples from HRT-18G cells was 15 258 times higher at 72 hpi than at 1 hpi (Figure 3A). In addition to the increase in viral RNA, an increase in viral titers was observed in samples collected from the EIEs (Figure 3B). However, the titers in samples from the ileal EIEs were peaked at 48 hpi and relatively decreased by 72 hpi. The viral load in samples from HRT-18G cells increased more substantially than those from EIEs. Viral proliferation was also evaluated by electron microscopy in monolayer EIEs derived from the jejunum. Virus particles were observed within the cytoplasm or cytoplasmic vesicles (Figures 4A and B). Moreover, numerous virus particles were identified on the cell membrane, and they were budding from the surface of the cell membrane and releasing into the extracellular space (Figures 4C–E).

## Discussion

We cultured equine intestinal crypts of the small intestine (duodenum, jejunum, and ileum) following the method used with human and mouse tissues [13]. The crypts formed 3D cell structures and could be passaged at least 15 times without visible morphological changes. IHC confirmed that the generated EIEs were composed of a large number of undifferentiated stem cells. This result is reasonable given that the culture medium used in this study contained growth factors intended to maintain the stem cell niche, including Wnt3a, R-spondin, and Noggin. In addition, differentiated enterocytes, enteroendocrine cells, goblet cells, and Paneth cells were identified. Thus, all cell types expressed in the small intestinal epithelium in vivo were identified in the EIEs. The origin of previously reported equine small intestinal enteroids was limited to the jejunum [14, 15, 19]. Our results demonstrated that EIEs can be generated from all three regions of the equine small intestine, including the duodenum, jejunum, and ileum. However, the EIEs in our study may have not completely recapitulated the cellular composition of the small intestinal epithelium in vivo, because stem cells originally located in the intestinal crypt bases were most representative (Figure 2). The differentiation of intestinal enteroids can be accelerated by reducing or removing Wnt, R-spondin, and Noggin in human and murine enteroids [12, 13]. Although we tried this, the proliferation rate was declined (data not shown), so we did not use the medium for differentiation in this study. Most recently, appropriate culture conditions that promote the differentiation of enteroids from the equine jejunum and colon have been reported [19]. The differentiation of EIEs should make it possible to recapitulate in vivo cellular composition more accurately and advance further research of host–virus interactions.

Intestinal enteroids that form 3D structures following cellular polarity face their apical surface to the inside of the structure, making it challenging to expose their luminal side to experimental agents. In several studies, intestinal enteroids were mechanically sheared to expose the luminal surface and inoculated with viral pathogens [10, 20]. However, there is a concern that this method does not accurately recapitulate the in vivo situation, as the basal side of cells is also exposed. By contrast, a 2D monolayer model in which enteroid-derived cells are seeded on flat-bottomed plates allows only the apical surface to be exposed to experimental agents. This approach is therefore commonly preferred in studies of host–virus interactions in humans and porcine [9, 21, 22]. Here, we used this approach to inoculate EIEs with ECoV. EIEs derived from any tissue (duodenum, jejunum, or ileum) could be transferred into a 2D monolayer. The cell density reached > 80% after 3–4 days in monolayer culture and the cell confluency was maintained during the study period. Therefore, a cell concentration of approximately 1 × 10^4^ cells/cm^2^ should be the most appropriate for monolayer culture in our protocol. Although each cell type was identified by IHC in the 3D EIEs, the cell composition was not characterized after monolayer culture. It is therefore possible that the monolayer model employed in the current study may have lacked some of the characterization present in the 3D model. This is likely to be a limitation of this study. The increase in ECoV RNA and viral titer in the samples from EIEs indicates that ECoV can replicate and maintain its infectivity in the EIEs. In addition, electron microscopy revealed the presence of virus-like particles in the cytoplasm, and a considerable number of viruses were budding from the cell membrane of the jejunal EIEs. These findings indicate that the viruses replicated inside the cell and were released from the cell membrane into the extracellular space. The viral titers in samples from ileal EIEs were declined at 72 hpi compared to 48 hpi, whereas the number of viral RNA showed a consistent increase up to 72 hpi. The reason for this discrepancy remains unclear. Although the cell density had reached over 80% at the time of ECoV inoculation in each monolayer EIEs, the number of cells and the cell viability were not evaluated. It is possible that changes in cell number or cell viability over time may have contributed to these results. Nevertheless, our study shows that ECoV can infect EIEs derived from all three regions of the small intestine and replicate in them. To our knowledge, this is the first report describing enteric virus infection in EIEs. Our previous experimental challenge study in horses showed that ECoV infected throughout the small and large intestinal tissues, as confirmed by qRT-PCR and in situ hybridization [1]. The current study reproduced the in vivo situation, suggesting that EIEs offer a promise as a novel in vitro model for studying ECoV infection. It was shown that ECoV more substantially replicated in HRT-18G cells and maintained its infectivity. The inoculated ECoV, NC99 strain, had been passaged multiple times in HRT-18G cells prior to the current study, which may have resulted in viral adaptation to HRT-18G cells and led to an active viral replication in them. A notable discrepancy was observed in viral titers between samples from HRT-18G cells and EIEs. These findings indicate that HRT-18G cells are more suitable for the proliferation of ECoV. However, it should be noted that HRT-18G cells are derived from human cells and are therefore unable to fully reproduce the biological response that occurs in equine intestinal epithelium in vivo. In contrast, EIEs are derived from host equine tissues that are naturally susceptible to ECoV infection and comprise various cell types that are expressed in the intestinal epithelium. Therefore, although infectious viral replication is more active in HRT-18G cells, EIEs are likely to be a more suitable model for investigating the host-virus interactions than HRT-18G cells. In humans and other animals, the potential applications of intestinal enteroids include the identification of the target cells of enteric pathogens, the elucidation of infectious mechanisms, and the isolation of viruses from clinical specimen [8, 9, 23]. To advance these studies, EIEs should be useful in vitro models.

Another limitation of the current study is that EIEs were generated from the tissues of only one horse. It is important to note that the intestinal enteroids originate from the donor’s tissues and therefore may exhibit characteristics inherited from those tissues. It is probable that enteroids derived from different individuals differ in susceptibility to enteric pathogens. For example, the growth rate of human rotavirus differed among intestinal enteroids derived from individual humans [23]. Therefore, it is preferable to generate EIEs from multiple individuals and compare their capacity to sustain viral infection in order to confirm whether our results are representative of EIEs in general.

In conclusion, we generated the duodenal, jejunal, and ileal EIEs from horse tissues and demonstrated that ECoV infects and replicates in them in a 2D monolayer model. EIEs offer promise as a robust tool with which to investigate interactions between the equine intestinal epithelium and ECoV in vitro.

## Data Availability

The datasets used and analyzed in the study are available from the corresponding author on reasonable request.
